# Renewed Risk for Epidemic Typhus Related to War and Massive Population Displacement, Ukraine

**DOI:** 10.3201/eid2810.220776

**Published:** 2022-10

**Authors:** Paul N. Newton, Pierre-Edouard Fournier, Dennis Tappe, Allen L. Richards

**Affiliations:** University of Oxford, Oxford, UK (P.N. Newton);; Mahidol–Oxford Tropical Medicine Research Unit, Bangkok, Thailand (P.N. Newton);; Institut Hospitalo-Universitaire Méditerranée Infection, Marseille, France (P.-E. Fournier);; Bernhard-Nocht-Institut für Tropenmedizin, Hamburg, Germany (D. Tappe);; Allen L. Richards Consulting, Damascus, Maryland, USA (A.L. Richards)

**Keywords:** typhus, *Rickettsia prowazekii*, *Pediculus humanus*, epidemic typhus, Ukraine, Russia, war, bacteria, vector-borne infections

## Abstract

Epidemic typhus, caused by *Rickettsia prowazekii* bacteria and transmitted through body lice (*Pediculus humanus corporis*), was a major public health threat in Eastern Europe as a consequence of World War II. In 2022, war and the resulting population displacement in Ukraine risks the return of this serious disease.

The war in Ukraine has produced devastation in the region unseen since World War II. Epidemic typhus, one of the diseases that ravaged Europe during that period and before, but nearly forgotten in 2022, risks returning because of war and massive population displacement. History suggests that planning is needed to prevent this disease from aggravating the current war-induced public health crisis. Epidemic typhus (also called louse-borne typhus) is caused by *Rickettsia prowazekii* bacteria and is transmitted through the feces of body lice (*Pediculus humanus corporis*) that live in clothes. Before the advent of antibiotics, mortality rates from epidemic typhus reached 60%, especially in persons who were elderly and malnourished. The disease can be reactivated, in the absence of lice, after many decades as Brill–Zinsser disease, which can lead rapidly to further epidemics if patients become infested with body lice (*1*).

Epidemic typhus is associated with war, poverty, homelessness, cold weather, crowding, unsanitary conditions, and refugee camps. The disease has generated very little recent public awareness; the most recent regional outbreak reported in the public domain in English occurred in Russia in 1997 (*2*).

During World War II, Ukraine and adjacent countries were ravaged by epidemic typhus, especially the Jewish populations who were forced into ghettos. The city of Lviv in western Ukraine was a center for typhus vaccine research, especially through the work of Fleck and Weigl (*3*). Ukraine now has an estimated 1.8 million persons >80 years of age (*4*), some of whom may have contracted *R. prowazekii* infection during the 1940s and are at risk for Brill–Zinsser disease. Body louse infestations among refugee and sheltering populations, living in overcrowded and unsanitary conditions because of war, may trigger epidemics of *R. prowazekii* infection. A further risk for these populations is infection with *Bartonella quintana* bacteria, the cause of trench fever, that is also transmitted by body lice.

The public health services of Ukraine and the Eastern Europe region face multiple threats. Does the current epidemic typhus risk warrant timely surveillance to curtail outbreaks? Public health organizations, including and organizations caring for refugees, might consider sending body lice specimens collected from patients for *R. prowazekii* PCR testing (a list of laboratories that can perform this test is available from the authors) to give early warning of outbreaks. When body lice are detected, these organizations could consider community treatment, including delousing and the administration of ivermectin (*1*,*5*) and doxycycline, while assays are performed.

Because PCR testing and tetracycline drugs are now available, we can respond in such dire circumstances to prevent *R. prowazekii* outbreaks before they occur. Public health officials could institute a system analogous to that for surveillance and control of plague and fleas. We now have the tools and treatments that can make it possible to avert and mitigate epidemic typhus outbreaks.
